# Supplementary material for: Are carbon emissions trading and green financial instruments synergistic? -Comprehensive quantitative research based on content analysis
Source: PLoS One. 2024 Mar 7;19(3):e0298601. doi: 10.1371/journal.pone.0298601 (PMC10919601; doi:10.1371/journal.pone.0298601)
Supplement: S1 Data — (ZIP) [file pone.0298601.s001.zip › Data/地级市Z_F工作报告.zip!/░▓╗╒/║╧╖╩╩╨/└┤╫╘╣1⁄2╓┌║┼í╛┬φ┐╦╩2╛▌═°í┐_2016.docx]

# **2016年政府工作报告**

发布时间：2016-01-18 15:05

【字体：[大](http://www.hefei.gov.cn/public/1741/javascript:void(0);) [中](http://www.hefei.gov.cn/public/1741/javascript:void(0);) [小](http://www.hefei.gov.cn/public/1741/javascript:void(0);)】

各位代表：

   现在，我代表市人民政府向大会报告工作，请予审议，并请政协委员和其他列席人员提出意见。

   一、跨越赶超的“十二五”

   “十二五”时期是合肥发展历程中波澜壮阔的五年。在省委省政府和市委的坚强领导下，我们深入贯彻党的十八大和十八届三中、四中、五中全会精神，积极应对复杂多变的宏观环境，强化创新驱动，聚焦调整转型，深化改革开放，建设生态文明，改善民生保障，全面完成了“十二五”规划确定的目标任务，“新跨越、进十强”逐步成为现实，建设长三角世界级城市群副中心迈出坚实步伐，打造“大湖名城、创新高地”显现崭新形象。

   ——过去五年，是综合实力大跨越的五年。主要经济指标年均“两位数”增长，总量位次在全国省会城市中不断前移。地区生产总值连跨三个千亿台阶、达到5660.3亿元，占全省比重由21.9%升至25.7%。财政收入突破千亿元、达到1000.5亿元，年均增长14.5%，其中地方财政收入达到571.5亿元、年均增长15.4%。全社会固定资产投资累计完成2.36万亿元、年均增长19.6%。规模以上工业总产值达到9312.8亿元，是“十一五”末的2.5倍；增加值2255.7亿元、年均增长15.6%；新增产值超百亿企业10户。社会消费品零售总额达到2183.7亿元、年均增长16.9%。城镇居民人均可支配收入达到32080元，农村居民人均可支配收入达到15890元。金融机构本外币各项存、贷款余额双双突破万亿元大关。

   ——过去五年，是创新发展大提升的五年。战略性新兴产业产值达到2788.8亿元、年均增长30.5%。新型显示、机器人列入国家区域集聚发展试点，集成电路、智能语音、太阳能光伏、新能源汽车、公共安全、生物医药、燃气轮机等保持国内领先。累计完成技改投资5265.4亿元、占工业投资61.2%，平板显示及电子信息、家电、装备制造产值均超千亿元，家电“四大件”产量稳居全国之首。服务业增加值达到2298.9亿元，电子商务、服务外包、旅游会展、新兴文化产业等现代服务业和研发设计、检验检测等高技术服务业发展迅猛。进入国家系统推进全面创新改革试验，全社会研发投入占GDP比重由2.1%升至3.2%，专利授权量年均增长40.2%，技术交易合同额年均增长28.7%，国家级高新技术企业数达到1056户、增长1.4倍，高新技术产业增加值达到1280亿元、占GDP23%，自主创新主要指标全部进入全国省会城市“十强”；合肥综合性国家科学中心积极筹建，中科大先研院等新型协同创新平台加速建设。四城区加快打造全省“首善之区”和辐射源，四大开发区奋力打造创新发展主引擎和新高地。五县（市）着力打造新型工业化主战场和城乡统筹新典范，综合实力全部进入中部百强，肥西、肥东跻身全国百强。农业转型成效明显，特色高效农业产值比重超过80%。

   ——过去五年，是改革开放大突破的五年。积极打造全面深化改革先行先试“合肥版”，69项改革纳入国家试点。顺利完成庐江矾矿等国企改革，整合形成基础设施建设、产业、地方金融三大市级国资控股平台。民营经济比重提高到53%，各类市场主体由17万户增至44.7万户。全面推进全口径预算、开门办预算和绩效预算，稳步推行“营改增”。构建扶持产业发展“1+3+5”政策体系，累计投入资金126.3亿元。建立和完善“借用还”一体化投融资管理体制，金融体制改革创出“一通三改”新模式，境内上市企业增至34家、居全国省会城市第7位。完善集体决策、“净地出让”、“双向约束”等制度，国家节约集约用地试点市建设成效显著。省市共建招投标平台，公共资源交易“合肥模式”领跑全国。全面推进农村土地承包经营权确权登记颁证试点，农业土地流转面积突破300万亩，新型农业经营主体达到6678家。文化体制改革取得突破，走出了一条公益性文化单位市场化、社会化运营管理的新路子。医药卫生体制综合改革深入推进，基层医改和县级公立医院改革基本完成。积极承接国内外产业转移，累计招商引资1.27万亿元、年均增长15.7%，其中外资95亿美元、年均增长13.2%，境外世界500强企业增至39家。多层次多领域拓展国际合作，不断巩固友城基础，对外贸易持续扩大，“四港三区一中心”等八大开放平台建设取得重大进展，成为“一带一路”和长江经济带双节点城市。对台经贸和交流合作不断深化，重大台资项目引进实现突破。积极与长三角、京津冀、珠三角、长江中游城市群等加强合作交流，充分发挥合肥经济圈核心带动作用，与皖北结对合作深入推进，援疆、援藏工作取得新成效。

   ——过去五年，是城乡面貌大变化的五年。顺利完成行政区划调整，大力实施“1331”空间发展战略。城市建成区面积扩大到416平方公里，常住人口增至409万，城镇化率提高到70%。中心城区功能优化，政务文化新区基本建成，滨湖新区快速崛起，各大组团不断拓展。大建设累计续建、新建工程1721项，完成投资1337.5亿元。全国性综合交通枢纽加快建设，新桥国际机场建成通航，高铁南站投入运行，“米”字型高铁网络初步形成，合肥港通江达海能力得到提升。全市公路总里程接近2万公里，“一环八线”高等级公路网基本形成，国省干道建设成为全国样板。轨道交通1、2、3号线加快建设，4、5号线规划获批。快速高架桥网络基本建成，公交、水电气热、通讯等公用事业保障有力。坚持“三城同创”，出台《城市管理条例》，成立城市管理委员会，推行网格化、精细化管理，成功跻身全国文明城市行列。以新型城镇化为引领，提升县城、新市镇、环湖十二镇等规划建设水平。全面加强美好乡村建设，深入推进以“三线三边”为重点的全域环境综合整治，累计建成省级示范村130个，整治自然村2000多个。耕地连续17年“占补平衡”，粮食生产“十二连丰”，粮库扩容110万吨，肉蛋奶水产品总产量105万吨，粮食安全得到有效保障。实施各类水利工程项目1.5万个，农村饮水安全工程覆盖所有乡镇。

   ——过去五年，是生态建设大推进的五年。巢湖流域跻身首批国家生态文明先行示范区，与国开行合作环巢湖地区生态保护与修复工程四期项目深入实施，120多条河道纳入“河长制”管理，城市污水集中处理率达到98%，环湖32座乡镇污水处理厂建成试运行，巢湖湖区总体水质由Ⅴ类好转为Ⅳ类。强力推进大气污染防治九大专项整治，PM10年均浓度由113微克/立方米下降到91.9微克/立方米，空气质量优良率达到69.9%。实施固体废弃物综合整治行动，推进生活垃圾焚烧、餐厨垃圾无害化处理和综合利用，肥东、长丰、庐江静脉产业园加快规划建设。大力推进“五森”工程，累计植树造林117.8万亩，全市森林覆盖率由20.5%提高到26.8%，城市建成区绿化覆盖率由44.4%提高到46%，成功创建国家森林城市。

   ——过去五年，是社会民生大改善的五年。率先出台基本公共服务体系规划和社会服务“1+4”政策，各项民生工程投入350亿元，惠及城乡居民3000万人次以上。实施“百村万户”精准扶贫工程，在全国首创“光伏扶贫”模式，贫困人口减少30万。坚持以创业带动就业，累计新增城镇就业岗位83万个，城镇登记失业率控制在4%以内。社会保障不断提标扩面，养老保险、被征地农民保障等实现全覆盖。建设各类保障性安居工程26万套，实施城中村、危旧房改造项目124个，整治老旧小区297个，城镇居民人均住房面积由26.3平方米增至35平方米。全面实施义务教育“三大提升”工程，新建和改扩建中小学校106所、幼儿园235所，全国义务教育发展基本均衡县（区）实现全覆盖。合肥学院成为全国应用型大学建设和改革的范例。新增医疗卫生服务机构1300家、床位1.2万张，基本建成覆盖城乡的基层医疗卫生服务体系。文化、体育、旅游等融合发展，渡江战役纪念馆、安徽名人馆等公益文化设施建成开放，大力推进省市共建美术馆、科技馆、规划馆等建设，成功举办环湖国际马拉松赛、中日韩三国围棋名人混双赛等品牌赛事和“青春毅行”等重大活动。《合肥市志》出版发行，《合肥通史》编纂完成。创新社会治理机制，社会组织覆盖面逐步拓展，志愿者队伍不断壮大，成为全国社会组织建设创新示范区。坚持用群众工作统揽信访工作，信访渠道进一步畅通。应急管理机制逐步健全，防空防灾一体化建设加快推进，救助救灾体系不断完善。严格落实安全生产责任制，烟花爆竹全行业退出和油气管线隐患整治等成效显著，各项事故指标逐年下降。国防动员和后备力量建设取得新进展，实现全国双拥模范城“七连冠”。扎实开展平安合肥建设，“六五”普法取得新成效，社会大局保持和谐稳定，连续第3次捧得“长安杯”。人口计生、民族宗教、外事侨务、防震减灾、统计、科普、气象、档案、保密等工作取得新成绩，工会、共青团、红十字会、妇女儿童、老年人、残疾人和关心下一代等工作实现新进步。

   过去五年，我们高度重视加强政府自身建设，政府治理体系不断完善，治理能力进一步提升。推进依法行政，健全完善政府重大行政决策程序，行政权力运行进一步规范透明，荣获“中国法治政府”提名奖。严格执行人大及其常委会的决议决定，自觉接受人大监督；主动接受政协民主监督，虚心接受社会舆论监督；办理人大代表议案和建议953件、政协提案2915件。改进工作作风，深入开展党的群众路线教育实践活动和“三严三实”专题教育，严格执行中央“八项规定”、国务院“约法三章”和省市相关规定，大力精简会议文件，全面改进文风会风，严格控制各类检查评比表彰，“三公”经费支出逐年下降。加强效能建设，大力实施简政放权，新一轮政府职能转变和机构改革全面完成，权力、责任、涉企收费“三项清单”制度全面推行，市级权力事项保留1457项、精简率73.7%，成为全国审批事项最少的省会城市之一。坚持廉洁从政，严格落实党风廉政建设责任制，全面加强惩治和预防腐败体系建设。推行审计监督全覆盖，加大对重大项目、民生工程、“三公”经费和违规收费等监督检查力度，深入推进政务公开、政风评议。

   各位代表！

   刚刚过去的2015年，我们坚持稳中求进、稳中强基、稳中创优，经济增长逆势上扬，社会发展平稳健康，较好地完成了去年初人代会确定的目标任务，为“十二五”收官划上了圆满句号，为“十三五”启航奠定了坚实基础。全市生产总值增长10.5%；规模以上工业增加值增长11.3%；财政收入增长13.6%，其中，地方财政收入增长14.2%；全社会固定资产投资增长15.4%；社会消费品零售总额增长12%；预计城镇居民人均可支配收入增长9.5%左右，农村居民人均可支配收入增长10.5%左右；城镇登记失业率控制在3%以内；居民消费价格涨幅1.6%。

   一年来，我们主要做了以下工作：

   一是千方百计稳定增长。积极应对经济下行压力，及时推出促进经济平稳较快发展30条、扶持小微企业健康发展18条、困难企业帮扶10条等政策“组合拳”，努力实现实体经济发展稳中向好。实施项目建设“十大行动”，积极对接国家重大工程包和专项建设资金，集中开展项目签约、开工活动，着力扩大有效投入。全年新开工项目6718个、增加1952个，京东方10.5代线、晶合12吋晶圆、大陆轮胎二期、中盐红四方二期、神皖庐江电厂、北大未名生物医药产业园等开工建设，江淮松芝汽车空调、宝龙达笔记本电脑、安凯新能源汽车等建成投产。完善金融服务体系，广发银行、渤海银行等落户开业，发行“大湖名城”系列财政金融产品，新型政银担合作实现“两个全覆盖”，全年新增贷款1504.3亿元。积极推进上市公司融资、再融资，参与上市公司资产重组，富煌钢构、三和科技首发上市，新增新三板挂牌企业34家，直接融资1732.6亿元、增长153.4%。

   二是加快产业转型升级。认真贯彻省委省政府“调转促”的重大决策部署，制定加快创新转型升级发展的行动计划，大力培育增长新动能。新型显示、集成电路、智能语音、新能源汽车入选全省首批战略性新兴产业集聚发展基地，战略性新兴产业对规模以上工业增长的贡献率达到54%以上。积极落实国家“互联网+”行动计划，率先实施“万千百工程”，移动互联网、云计算、大数据、物联网等与制造业加速融合。出台促进旅游业、养老服务业发展等实施意见，现代服务业完成投资3022.8亿元、增长17.5%；获批首个环巢湖国家旅游休闲区，三河镇成功创建国家5A级景区；网络经营主体发展到7万户，一批商业综合体建成开业；承办和举办机器人世界杯赛、家博会、苗交会等特色大型展会181场。

   三是大力实施创新驱动。中科大先研院与英特尔、微软、阿里巴巴等联合共建研发平台36家、孵化科技企业136家，清华大学合肥公共安全研究院建成启用，与中科院合肥物质研究院合作共建离子医学中心，与中科大、安大分别谋划设立滨湖国际金融研究院和绿色发展研究院，支持中科大、合工大申办示范性微电子学院。入选国家小微企业创业创新基地城市示范，出台“三年行动计划”和28条“双创”政策，成功举办全国“发现双创之星”和全国“双创活动周”合肥分会场系列活动，建成5F创咖等众创空间24家、科技孵化器32家。成立机器人、集成电路、新能源汽车、轨道交通装备等产业技术创新战略联盟。新认定国家级高新技术企业363家，各类企业研发机构增至934家，开通网上技术交易平台。

   四是全面深化改革开放。政府投资引导基金母基金规模超过30亿元，参股设立子基金10余支，撬动社会资本近70亿元。商事登记实现“三证合一、一照一码”。市、县（市）区、乡镇（街道）三级政府权力清单和责任清单体系建成，事中事后监管进一步强化。启动聘任制公务员试点。市级公务用车改革基本完成。推进户籍制度改革，实施居住证管理办法。建立居民生活用气阶梯式价格制度，调整污水处理费、水资源费征收标准和城市供水价格。稳步实施村级集体资产产权股份合作制改革试点，加快推进农村新型流通体系建设。制定加快开放平台建设发展的实施意见，安徽（蜀山）跨境电子商务产业园正式运行，合肥港集装箱年吞吐量达到18万标箱，“合新欧”国际货运班列和合肥空港往返深圳全货运包机常态运营；对外劳务合作服务中心基本建成。积极与上海开展双城合作，加强与央企、知名民企、外资企业合作，全年招商引资3390亿元、增长15%，其中外资25.1亿美元，进出口总额达到203.4亿美元。

   五是积极推进城乡一体。加快编制新型城镇化、市政基础设施等重大规划。庐铜铁路加快建设，商合杭高铁、合安九客专开工建设。合肥火车站站前广场、绕城高速下穿南站南广场改建工程进展顺利，西客站站前广场工程完工，南薰门桥、望江路改造、繁华大道东延、花园大道等建成通车，巢湖南路、老合淮路等在建工程加快推进。续建、新建城市支路工程97项，完成勤劳巷等小街巷改造14个。滨湖新区集中供热工程建成运行。4个区域地下综合管廊样板工程试点稳步实施。开工建设停车场16处、1280个泊位。安排30亿元专项资金，加大县域基础设施建设支持力度。推进城乡园区共建和产业融合，肥西桃花工业园成为全省首个千亿元县域工业园。完成第二批82个省级美好乡村示范村建设和656个自然村整治任务。加强现代农业园区建设，新增家庭农场等新型农业经营主体1343户。实施100公里县乡公路升级改造和300公里提级联网延伸项目建设。气象灾害监测预警一期工程等建成使用。

   六是加强环境综合治理。制定水污染防治方案，严格落实排污口整治、生态补水、湿地净化等措施，完成42个老旧小区雨污分流改造，开展187个小流域污染源调查。开工建设引江济淮工程试验段，启动实施清溪净水厂项目，顺利通过国家最严格水资源管理考核和水生态文明城市试点建设实施方案评审。全年全域全面秸秆禁烧成效显著。加强农业面源污染治理，化肥、化学农药使用量同比分别下降6.6%、6.2%。淘汰黄标车44867辆，2005年底前注册营运黄标车全部淘汰。马（合）钢公司钢铁冶炼及长材生产线实现关停，基本完成市区燃煤及其他非清洁能源锅炉淘汰任务。完成植树造林16.8万亩、城区绿化1660万平方米，打造园林绿化精品示范工程28个。

   七是加大民生保障力度。编制实施城市基本公共服务设施专项规划，建立滚动公益性项目库。“32+9”项民生工程加快推进，各级财政投入82.3亿元。开发公益性岗位1万多个，高校毕业生进肥就业率达96.3%。降低职工医保缴费比例，统一居民医保参保政策，加强严重精神障碍患者救治救助工作。完善国有土地上房屋征收与补偿办法，出台公租房管理规定。加强中小学、幼儿园和职业院校教师队伍建设，完成义务教育学校标准化建设任务，十中新校区建成使用，组建现代职教集团，合肥职业技术学院、合肥幼儿师范高等专科学校获批全省地方技能型高水平大学立项建设。113个公共文化场馆免费开放，接待市民600多万人次。推进军民融合发展，加强与“合肥舰”双拥共建。食品药品重大事故“零发生”，各类安全生产事故指标创历史最低水平。规范信访事项受理办理程序，化解省交办信访积案118件。圆满完成全国第三次经济普查任务。

   各位代表！

   五年的成就令人振奋，五年的经验弥足珍贵。我们善谋大势，始终面向全国、放眼全球，积极顺应世界新一轮科技革命和产业变革的新趋势，主动适应我国经济发展新常态，加速融入国家区域发展新棋局。我们敢走新路，坚持解放思想，勇于先行先试，以敢下深水的决心，实施改革攻坚突破，推出了一大批示范全省、引领全国的创新力作。我们聚焦转型，瞄准国际国内前沿和高端，坚持创新转型升级发展不动摇，加快打造全国产业创新中心和世界级产业集群。我们强化统筹，坚持包容和共享，全面推进城乡融合发展、经济社会协调发展、人与自然和谐发展。我们简政敬民，始终以敬民之心行简政之道，大力打造法治政府，加快政府职能转变，全面提高服务效能。

   各位代表！

   “十二五”取得的巨大成就来之不易。这是省委省政府和市委坚强领导的结果，是市人大依法监督、市政协民主监督、社会各界大力支持的结果，是全市人民团结奋斗、顽强拼搏的结果。在此，我代表市人民政府，向辛勤奋战在各个领域、各个岗位为合肥发展做出贡献的全市人民，向驻肥人民解放军、武警官兵、公安干警和中央驻肥单位，向各民主党派、工商联、无党派人士、各人民团体和社会各界人士，向所有关心支持合肥改革开放与现代化建设的海内外朋友，表示衷心的感谢和崇高的敬意！

   在肯定成绩的同时，我们也要清醒地看到，发展中还存在许多不足和问题，主要是：城市综合实力不够强，经济结构不够优，产业层次不够高，特别是第三产业比重偏低，民营经济发展不充分，战略性新兴产业规模不够大；城区经济转型不快、县域经济发展不足，居民收入水平不高，城乡统筹、区域协调、包容共享任务繁重；资源环境约束趋紧，节能减排压力加大，巢湖综合治理、大气污染防治任重道远；改革攻坚难度加大，开放型经济发展水平不高，国际化水平较低；优质公共服务供给不足，城市管理精细化、专业化、现代化水平不高；法治政府建设有待加强，政府机关服务效能与群众期盼还有差距，不作为、慢作为、乱作为等现象还时有发生。对这些问题，我们要高度重视，采取切实有效措施，认真加以解决。

   二、创新转型的“十三五”

   “十三五”是合肥率先全面建成小康社会的决胜时期，是合肥加快创新转型升级发展的关键时期，我们既面临难得机遇，也面临严峻挑战，但仍处于大有作为的重要战略机遇期和黄金发展期，必须以全球视野、战略思维，在新一轮世界科技革命和产业变革中抢占先机、加快转型，在全国区域发展新棋局中提升能级、凸显地位，在安徽创新型“三个强省”建设中勇挑重担、示范引领，加快建设长三角世界级城市群副中心，打造提升“大湖名城、创新高地”。这是时代赋予我们的历史使命，是省委省政府授予我们的政治责任，是全市人民寄予我们的热切期待。只要我们把握机遇、主动作为、精准施策，就一定能够把合肥建设和发展推上新的更高水平！

   今后五年，经济社会发展的指导思想是：高举中国特色社会主义伟大旗帜，全面贯彻党的十八大和十八届三中、四中、五中全会精神，认真落实“四个全面”战略布局，牢固树立“五大发展理念”，深入贯彻省委、省政府决策部署，坚持创新转型升级发展不动摇，以提高发展质量和效益为中心，以加快调结构转方式促升级为总抓手，以增进人民福祉、促进人的全面发展为出发点和落脚点，统筹推进经济建设、文化建设、社会建设、生态文明建设和法治政府建设，当好全省“三个排头兵”，确保率先全面建成小康社会，加快建设长三角世界级城市群副中心，进一步开创“大湖名城、创新高地”建设新局面。

   今后五年的主要奋斗目标是：经济总量位次前移，经济增速力争“两位数”，保持全国省会城市前列；总量位次稳居全国省会城市“十强”，并力争前移。到2020年，经济总量冲刺10000亿元，人均GDP达到12万元；财政收入力争1600亿元，其中地方财政收入力争840亿元；规模以上工业增加值达到4000亿元，服务业增加值达到4600亿元；累计完成全社会固定资产投资4万亿元；社会消费品零售总额达到3500亿元。创新水平全国一流，建设具有国际影响力的创新高地，全社会研发投入占GDP比重达到3.5%，高新技术产业增加值占GDP比重达到25%，专利授权量和技术交易合同额总量翻一番、年均增长15%，国家级高新技术企业达到1600户。万元生产总值能耗、主要污染物排放总量等节能减排指标完成省控目标。城市功能显著增强，城镇化率超过75%，市区常住人口突破500万人，建成区面积扩大到500平方公里，轨道交通、水电气热、公交、通讯等公用设施不断完善，全国性综合交通枢纽地位进一步彰显，国际化都市区框架加快形成。民生福祉持续改善，居民人均可支配收入突破4万元，城镇登记失业率控制在4.5%以内，城乡养老保险、医疗保险实现全覆盖，城乡居民最低生活保障水平全面并轨，建成保障性安居工程13.6万套，贫困人口全面脱贫，普及高中阶段教育，医疗机构每千人床位数超过8.5张，全民健身活动广泛开展，大力建设健康合肥。文明程度明显提高，中国梦和社会主义核心价值观更加深入人心，社会文明风尚更加浓厚，文明城市建设深入推进，市民思想道德素质、科学文化素质等明显提高。公共文化服务体系全面建成，文化产业成为重要支柱产业，城市品牌形象唱响中国、走向世界。全社会法治意识不断增强，平安合肥、法治合肥建设全面推进，社会治理水平明显提升，社会更加和谐稳定。生态环境不断优化，巢湖生态文明先行示范区建设取得重要进展，巢湖水质总体达到地表Ⅳ类水标准，城市污水集中处理率达到98%，垃圾无害化处理率、工业固体废物处置利用率均达到100%，森林覆盖率超过28%，城市建成区绿化覆盖率达到46%，非化石能源占一次性能源消耗比重超过8%，空气质量优良率进一步上升。体制机制更加健全，城乡治理体系和治理能力现代化取得重大进展，全面创新改革试验率先突破，全面深化改革走在前列，法治政府基本建成。内陆开放高地建设取得重大进展，开放平台体系和功能更加完善，国际化及区域合作水平明显提升，累计招商引资2万亿元、利用外资180亿美元，进出口总额达到300亿美元。

   今后五年，我们要重点在五个方面实现新突破：

   坚持创新发展，进一步培育转型升级新引擎。创新是引领发展的第一动力。深入实施创新驱动发展战略，系统推进全面创新改革试验，基本建成合肥综合性国家科学中心和全国性产业创新中心。支持中科院合肥物质研究院、中科大等建设超导核聚变中心、中国量子中心、空地一体化网络等一批国家级重大工程平台，打造联合微电子中心、离子医学中心、分布式智慧新能源集成创新平台等一批全国性产业创新中心，推进中科大先研院等10个以上新型协同创新平台建设，形成10个以上国家级和省级战略性新兴产业集聚发展基地，建设国际一流的新型显示产业集聚区、世界级光伏产业集群，打造中国IC之都、“中国声谷”和全国重要的新能源汽车产业集群、离子产业集群、智能制造产业集群、生物产业集群，千亿元战略性新兴产业基地达到4个。落实国家“互联网+”行动计划和“中国制造2025”战略，推动传统制造业向柔性、智能、精细转变。以建设服务业集聚区为载体，以壮大高技术服务业为重点，实现服务业发展提速、比重提高、水平提升。

   坚持协调发展，进一步构筑经济发展新格局。协调是持续健康发展的内在要求。坚持区域协同、城乡一体，全面优化都市区功能布局和空间形态，形成主城区、副中心、新市镇、美丽乡镇协调发展的新型城镇化格局，持续加大城乡基础设施建设力度，不断增强城市综合承载力和辐射带动力。城区全力打造国际化都市区的核心区、高端服务业的集聚区，开发区全力打造创新创业的引领区、产城融合的示范区。实施县域经济突破工程，力争五县（市）全部进入全国百强县行列。落实长江经济带综合立体交通走廊规划，建成全国最发达的铁路枢纽之一、内陆地区重要的集装箱中转枢纽港和江淮航运中心。稳步提升城乡要素供给能力，促进城乡公共资源均衡配置。规划建设国家级合肥滨湖新区。

   坚持绿色发展，进一步厚植生态文明新优势。绿色是永续发展的必要条件和美好生活的重要体现。构建政府、企业、公众共治的环境治理体系，实行最严格的环境保护制度，打造天蓝、地绿、水清、山美、城靓的美好家园。突出生态优先、城湖共生理念，继续推进巢湖综合治理等工程建设，实施全市域小流域“山水田林路村园”综合整治，实现巢湖和入湖主要河流水质明显改善。巩固“九大行动”成果，持续改善空气质量。积极发展全域旅游，高质量开发湖光山色和温泉、湿地、历史文化等资源，全力推进环巢湖国家旅游休闲区建设。巩固扩大国家森林城市创建成果，积极创建国家生态园林城市。大力倡导绿色、低碳、循环发展，加快推动生产方式、生活方式和消费模式绿色化转型。

   坚持开放发展，进一步打造对外开放新高地。开放是繁荣富强的必由之路。发挥“一带一路”、长江经济带双节点城市功能，坚持内外需协调、进出口平衡、引进来和走出去并重、引资和引技引智并举，统筹国际国内“两个市场”和“两种资源”，对接高新技术和社会资本，推动产业升级。高水平建设“四港三区一中心”等重大开放平台，编制实施城市国际化行动纲要，打造开明开放、接轨国际的内陆开放高地。全面融入长三角，主动对接京津冀、珠三角，推动合肥上海双城合作，强化与武汉、长沙、南昌在基础设施、公共服务、社会保障等领域互联互通互认。加快合肥经济圈向合肥都市圈战略升级，打造具有全国影响力的都市圈品牌。全力推动空港经济示范区建设。支持阜阳合肥、寿县蜀山、临泉庐阳等现代产业园区建设上规模、见成效。

   坚持共享发展，进一步增强人民生活新福祉。共享是中国特色社会主义的本质要求。按照人人参与、人人尽力、人人享有的要求，推进改革发展成果更多更公平地惠及广大人民，打造更具获得感的幸福城市。坚持居民收入增长和经济增长同步，多渠道增加居民工资性、经营性、财产性、转移性收入。加快完善就业创业、社会保障、国民教育、医疗卫生、养老健康、住房保障等服务体系，促进基本公共服务均等化、优质化。大力发展文化事业，创建国家公共文化服务体系建设示范区，推动文化与科技、教育、旅游、金融、建筑等深度融合，打响合肥文化品牌。深入贯彻落实全民健身国家战略，打造一系列影响力大、群众参与度高的体育赛事。推进社会治理现代化，着力构建公共安全体系，全力维护社会和谐稳定。

   三、奋力前行的2016年

   2016年是实施“十三五”规划的第一年，我们要认真贯彻中央和省里的决策部署，主动适应新常态，积极应对下行压力，坚持稳中求进工作总基调，按照“三去一降一补”要求，突出供给侧结构性改革，强化底线思维，保持战略定力，勇于攻坚克难，创造性地做好工作，确保实现“十三五”发展良好开局。今年经济社会发展主要预期目标是：全市生产总值增长10%左右，全社会固定资产投资增长11%，财政收入和地方财政收入分别增长9%、8%左右，社会消费品零售总额增长11%，居民人均可支配收入力争增长10%。

   今年，要努力做好十个方面工作：

   （一）着力推进实体经济稳定发展。

   加大企业帮扶。认真落实国家、省各项支持政策，加快制定我市针对性、精准性、实效性更强的具体政策措施。修订完善扶持产业发展“1+3+5”、困难企业帮扶10条等政策，加大兑现密度，简化办事流程。坚持分类施策、有进有退，对产品有市场、有效益但暂时遇到困难的企业予以支持，对停产半停产、连年亏损、资不抵债的企业通过实施兼并重组、破产关闭等办法，盘活存量资产。积极开展降低实体经济企业成本等专项行动，加大结构性减税和普遍性降税减费力度，坚决清理各种不合理收费特别是垄断性中介服务收费。全面做好地产品产销衔接，大力帮助企业开拓国内外市场。

   稳定房地产市场。坚持供需两个方向发力，多措并举促进房地产市场健康发展。建立购租并举的住房制度，加大保障性安居工程建设力度，完善廉租房、公租房并轨运行，积极推行货币化安置和先建后拆，新建棚户区改造安置房2万套以上。加大进城农民购房贷款支持力度，推进非公企业建立住房公积金制度。分类确定土地供应规模，把握土地供应节奏，改善房地产供给结构，有效释放住房刚性需求和改善性需求。

   加强融资服务。依托地方国有控股平台，加快政策性融资担保体系建设，深化政银担风险分担机制。充分发挥财政杠杆作用，设立转贷资金，创新金融产品，引导银行机构加大信贷投放。健全政府投资引导基金运作机制，推动股权投资基金集聚发展。规范互联网金融，坚决遏制非法集资。抓住股票发行注册制改革、战略性新兴产业板设立等机遇，支持企业首发上市、在新三板和区域股权交易市场挂牌，鼓励企业参与上市公司重组。引导企业发行公司债、私募债，运用好集合债等金融工具。

   强化要素保障。推进三水厂迁建、七水厂二期和巢湖三水厂建设，实施供水管网互联互通工程。新建肥北500kV等电力工程30项、总投资19亿元。加快天然气高压管网建设，大力发展CNG常规站等新型工业用户，进一步增强供气保障和储气调峰能力。开工建设金源热电2×350MW改扩建工程。

   （二）着力推进转型升级。

   集聚发展新兴产业。突出十大重点产业，加快建设战略性新兴产业国家集聚发展试点和首批4个省级集聚发展基地，谋划申报新一批省级基地，梯度建设一批市级基地。加快推进通富芯片封装、射频芯片研发等重大项目建设，推动江淮电动轻卡、中航新能源汽车、国轩动力电池、阳光三星储能等项目试产投产。积极布局未来型先导产业，推进燃气轮机、数控机床、机器人等一批前瞻性重大项目建设。加快国际智能语音产业园等建设，全力创建“中国软件名城”。修订完善推广应用政策，推动光伏电站资产证券化和产业集聚，申报建设国家光伏“领跑者”计划示范基地。

   改造提升传统产业。加快“两化”深度融合，推进传统产业高端化、智能化、绿色化。综合运用财税、金融等政策，积极引导企业大规模开展技术改造和产品升级。实施“万千百”创新工程，大力推进惠而浦变频滚筒洗衣机、海尔智能工厂、长安自主轿车二期、合肥欧洲工业设计中心等重大项目建设。实施“机器换人”行动计划，进一步提升装备制造、家用电器、汽车及零部件、安全食品等优势传统制造业核心竞争力。推动主城区工业园区转型升级，打造若干综合效益明显的都市产业园区。支持瑶海老工业区加快搬迁改造。筹建全国再制造产品交易中心。

   加速服务业发展。围绕消费升级和新兴业态发展，突出现代物流、金融服务等十大重点服务业，谋划申报一批省级服务业集聚区，推进建设一批市级服务业集聚区。依托滨湖国际金融服务后台基地，积极引进相关金融企业，打造金融上下游产业链。大力引进国内外知名电商和快递物流、金融支付企业，加快建设国家电子商务示范城市。围绕全国服务外包示范城市建设，搭建国际研发服务外包创新平台，打造一批综合配套功能完善的服务外包园区。出台支持政策和服务目录，引导检验检测产业集群集聚发展。加强现代物流园区和物流平台规划建设，谋划实施综合物流基地和环状快递产业园等项目。做大做强会展业，精心举办家博会、文博会、新能源汽车展、工业设计大赛、工业4.0峰会、海峡两岸半导体论坛等展会和活动。加大资源整合力度，聚焦健康产业发展，推进健康合肥建设。加快全国旅游标准化城市试点建设，推动万达文化旅游城等重大项目建成开业。推进社会服务管理信息化，扩大信息消费和信息服务。

   （三）着力推进自主创新。

   提升企业创新能力。支持企业建设工程（技术）研究中心、企业技术中心和重点（工程）实验室，引导龙头企业联合高校、科研院所建立产业技术创新战略联盟，争创2个国家级、40个省级企业技术中心和3个国家级、15个省级工业设计中心。组织实施爆发性增长源工程，完善科技型中小企业综合服务体系，支持企业引进关键技术。扩大高新技术企业认定范围，加强高新技术企业梯队培育。深化产学研用合作，围绕企业关键技术需求，积极与企业携手投入重大项目。大力实施“三年行动计划”，加快创建国家小微企业创业创新基地城市示范，支持高新区申报建设国家新兴产业“双创”示范基地。

   推进协同创新平台建设。加快启动建设合肥综合性国家科学中心和全国性产业创新中心，加强中科大先研院等新型协同创新平台和十个新兴产业研究院建设，推出一批突破性创新成果。完善平台管理办法，理顺资金、资产、产权关系，搭建技术资本对接和产业孵化的有效载体，促其早见成效。大力吸引海内外高端研发资源来肥设立全球领先的实验室和研发中心，支持高校院所、投资公司、龙头企业设立众创空间、建设孵化器，新建一批创新创业园。

   优化创新生态环境。实施系统推进全面创新改革试验，全面贯彻落实国家促进科技成果转化法，进一步优化科技成果转化、技术交易等政策，完善政府投资引导基金、天使投资基金风险容忍和尽职免责机制。建立知识产权运营中心，支持高校院所设立知识产权转移推广机构。积极引进海内外高层次人才和团队，支持科技人员创业创新，建立职务发明法定收益分配制度、科研项目间接费用管理制度和市场化薪酬制。大力弘扬创业创新文化，举办创业创新大赛，全面开展“四进四扶”活动，培植一批“双创之星”。

   （四）着力推进重点领域改革。

   深化经济体制改革。推进国有企业股权多元化改革，大力发展混合所有制经济，提升国有资本证券化水平，完善国有企业分类监管和县（区）国资监管体制。推行全面规范、公开透明预算制度，探索增强财政产业政策综合效应新路径。加强政府债务管控，创新政府投融资机制，加快推广运用政府与社会资本合作（PPP）模式。建立社会资本投资公共产品和服务项目建设的价格服务体系，放开一批政府定价和指导价审批项目。完善资源有偿使用制度和生态补偿改革措施。积极推进农村土地承包经营权确权登记，规范土地流转经营管理，完善农村产权交易机制。

   深化行政体制改革。完善“三项清单”制度，简化优化行政审批项目和流程，积极推进行政审批、公共服务事项网上运行和办理，做好取消和下放行政审批事项承接落实，健全事中事后监管制度，实现“放管服”有机统一。推进商事制度改革，确保完成18万户企业“三证合一”、“一照一码”换照工作。完善省市共建联管机制，在全省率先建成省市县三级一体化的公共资源交易平台。深化城市执法体制改革，开展县级重点领域综合执法试点，推进行政执法权向乡镇延伸。深化事业单位分类改革，推进法人治理结构试点。

   深化社会体制改革。创新社会治理模式，进一步理顺市、区、街道、社区管理职能。深化城市公立医院综合改革，全面落实国家基本药物制度。完善各项社会保险关系制度衔接与跨区域转移接续政策，发展职业年金、企业年金、商业养老保险，健全社会救助体系，积极推进医养结合。深化教育领域综合改革，推进教育管评办分离，建立学生学业质量绿色指标综合评价体系，探索中小学编制资源优化配置的有效途径。（下转3版）

   深化文化体制改革，加快发展新兴文化业态，推进传统媒体和新兴媒体融合发展。建立水环境常态治理机制，探索建立水权交易制度。健全资源节约和污染防治制度，积极开展排污权、节能量、碳排放交易试点。

   （五）着力推进对外开放。

   加快建设开放平台。完善合肥港国际航运和通关服务功能，加强与南京港、宁波港等合作，加快引进船代、货代公司，努力打造“一带一路”沿线重要节点港口。加密“合新欧”国际货运班列，推进市场化运行，建立物流双向机制。积极开通国际货运航线，建成合肥空港水果、冰鲜水产品进境指定口岸，启动粮食、药品、食用水生动物进境指定口岸申报。加快国家级跨境电商综合试验区建设，构建“六体系、两平台”，建成国际邮件互换局。加大综合保税区、出口加工区外向型重大项目招商和建设力度，力争空港保税物流中心（B型）尽快获批。

   大力推进招商引资。充分发挥“合肥之友”等平台作用，深入开展“县干大招商”等活动，修订完善大项目招商引资政策导则，着力营造亲商、安商、温商的良好环境，继续针对重点区域、重点产业、重点项目、重点企业，开展“一对一”、“点对点”招商，大力吸引跨国公司等在肥设立各类区域总部，力争全年招商引资3400亿元、利用外资28亿美元。积极支持优势企业走出去开展并购重组，抢占技术高地，开拓新兴市场。鼓励加大原材料、关键零部件、先进设备和群众需求大的日用消费品进口，推进外贸优进优出。

   深化国际国内合作。抓住中德合作契机，推进中德教育合作示范基地、德国创新产业园等项目建设。依托中俄“两河流域”和中美绿色环保等合作机制，推动更多项目取得实质性成果。拓展与“一带一路”沿线、中东、南美等国家和地区及友城的务实合作。全面推动与上海双城合作，深化与长江中游城市群联动发展。加快合肥经济圈领导会商会议定事项落实，深入开展与阜阳市及霍邱县、寿县结对合作，进一步延长和拓展支持阜阳合肥、寿县蜀山、临泉庐阳现代产业园区发展的政策。

   （六）着力推进都市区建设。

   优化空间布局。按照“一尊重、五统筹”的要求，进一步提升城市规划建设管理水平。推进“四规合一”，启动修编城市总体规划，加快编制主体功能区、都市区（圈）、全国性综合交通枢纽等规划。四个城区发展以国际化都市区为标杆，突出功能分区，加快实施“退二进三”。四大开发区着力打造高端要素的集聚区和辐射带动区域发展的主引擎，加快实施“优二兴三”。大力推进东部、南部、北部三大城市副中心建设，促进产城融合，实现宜居宜业。支持包河、蜀山经济开发区升级为国家级开发区。支持新站区申报国家产城融合示范区。积极申报建设国家级合肥滨湖新区。

   增强承载能力。推进骆岗机场复航及综合开发利用，探索发展地方通用航空。加快建设商合杭高铁、合安客专，建成庐铜铁路，完成宁西铁路增建工程，争取合肥至南京、合肥至青岛等快速客运铁路纳入国家规划，加快合肥至新桥机场至六安、合肥至巢湖至马鞍山城际铁路前期工作。大力推进合宁、合安、合巢芜高速公路“四改八”工程，全面建成合裕航道，完成派河、西河、兆河、白石天河等航道综合治理工程。建成运营轨道交通1号线，加快建设2号线、3号线，兴建4号线、5号线，启动新一轮轨道交通前期工作。开工建设合相路，加快建设合水路、合铜路二期及环巢湖道路各连接线，加快推进绕城高速下穿南站南广场、郎溪路高架建设，启动铜陵路高架北延、裕溪路高架东延等工程。加强小街巷改造，建设凤台路等一批支路。推进地下综合管廊和“海绵城市”建设。加大“公交都市”创建力度，建设公交专用道18条，新增和更新公交车600台。加强公共停车场建设，新建36处、5700多个泊位。

   提升城市品质。加强文明城市建设，巩固提升全国文明城市创建成果。推进信用城市建设，加快构建覆盖全社会的信用体系。整合信息资源，建设“智慧合肥”。拓展“数字城管”系统功能，提升数字化、动态化、实时化管理水平。开展25个老旧小区综合整治，新启动城中村、危旧小区改造项目10个，实施一批燃气配套和管道液化气改造等工程，提升物业管理和服务水平。加强城市设计和立面景观综合整治，加快特色街区和寿春路、宁国路、芜湖路、黄山路等精品道路建设。加强再生资源回收利用体系建设，积极开展生活垃圾分类收集，深入推进路面净化。

   （七）着力推进城乡统筹。

   加快做强县域经济。启动小流域综合治理，全面加强道路、电网、供水等农村基础设施建设，整体提高县域产业承载力。继续安排30亿元专项资金，支持县（市）大建设。在重大项目建设、招商引资项目布局、政府投资引导基金投入、产业政策支持等方面，加大对县域产业发展的倾斜力度。支持肥西桃花工业园、长丰双凤工业园升级为国家级开发区。实施新型城镇化规划，强化市与县（市）联动，加快推进与国开行等合作试点项目建设。整合各类资金50亿元，支持县（市）区社会事业发展和民生保障。继续加大对巢湖市、庐江县支持力度，促进县域经济协调发展。

   加快建设美丽乡镇。科学编制美丽乡镇建设规划，进一步优化美丽乡镇空间布局。按照中心城市标准，大力提升县城规划建设水平。完成34个乡镇政府驻地整治建设任务，突出抓好治脏治乱，强化基础设施建设和公共服务配套。以环湖十二镇等为重点，优选一批基础较好的乡镇，加快打造特色小镇。深化户籍制度改革，全面放宽城镇落户条件。推进37个中心村建设和一批自然村环境整治，分层分类打造美丽乡村“基本版”、“标准版”和“升级版”。支持巢湖市整市开展全省美丽乡镇建设试点。

   加快发展现代农业。实施现代农业“十大行动”，推动农业结构调整转型，加快发展规模农业、园区农业、设施农业、生态农业，继续在环巢湖地区推进化肥、农药减量使用。实施种业“百亿”提升工程，构建“育繁推”一体化的现代种业集群。推进农业经营体系建设，新增各类新型农业经营主体1000家。建设国家级农业气象试验基地，新增高标准农田5万亩以上。实施淠史杭、驷马山两大灌区续建配套和节水改造工程，完成71座中小型水库和2座闸坝除险加固，全面解决农村安全饮水问题。

   加快实施脱贫攻坚。围绕“一年脱贫攻坚、四年巩固提升”的目标，按照“政府主导、社会参与、精准到户、扶贫到人”的思路，坚持“开发式”扶贫与“兜底式”帮扶并举，专项扶贫、行业扶贫、社会扶贫联动，深入推进“十大扶贫到户到人”、“五大到村”、“三无特困农户政府兜底”三大精准扶贫攻坚工程，确保今年底现行标准下全市建档立卡的21.56万农村贫困人口全部脱贫，112个重点贫困村全部出列，在全省率先实现整市整体脱贫。

   （八）着力推进生态建设。

   强化巢湖综合治理。抓好引江济淮工程建设。加快实施环巢湖地区生态保护与修复工程，一期工程充分发挥效益，二期工程全面完工，三期工程全面开工，四期工程完成初步设计。大力开展南淝河、十五里河、派河综合治理，建成十五里河三期、蔡田铺二期、小仓房二期、西部组团等污水处理厂，加快建设清溪净水厂、胡大郢污水处理厂，新增城市污水日处理能力35万吨。加大水污染防治力度，完善“河长制”长效机制，推进城市黑臭水体整治，确保董铺、大房郢水库等饮用水源安全。

   创建国家生态园林城市。巩固国家森林城市创建成果，推进“五个一”绿化工程，完成江淮分水岭脊线区域、环巢湖沿岸、水源保护区等重点工程造林10.7万亩，实施一般成片造林2.3万亩。推进滁河干渠风光带规划建设，新建和完善森林长廊242.3公里。积极申报滨湖、三河、庐阳三处国家级湿地公园，创建省级森林城镇7个、森林村庄50个。新建和提升城区绿化1000万平方米，新增公园游园、街头绿地35个，进一步提升绿化管护水平。

   深化大气污染防治。坚持以“三厂两尘两气”为重点，大力推进大气污染联防联控。实施清洁能源普及计划，加快推进“气化合肥”，扩大光伏发电示范应用。强化工业废气治理，全面推进重点行业除尘和脱硫脱硝升级改造，对城区重污染企业实施搬迁改造。实施建筑垃圾、工程渣土全密闭运输和安全堆放，严控道路交通、施工工地、料场、裸露地面等扬尘污染，大力整治餐饮油烟污染。全面淘汰黄标车，鼓励购买使用新能源汽车。深入实施全年全域全面秸秆禁烧，完善长效管理和综合利用机制。加大大气监测力度，建立预警体系，多措并举有效缓解雾霾，确保完成省下达目标任务。

   （九）着力推进社会治理。

   实施民生工程。完善民生工程社会公开征集系统，精选惠民利民项目。加强民生工程资金统筹管理，提高使用效益。健全社会保障管理体制，大力整合城乡居民医疗保险制度，积极推进社会保险服务向基层延伸，建立健全社会保险公共服务体系。推进城镇职工医保大病救助和城镇居民医保购买商业大病保险。全面实施困难残疾人生活补贴和重度残疾人护理补贴。

   发展社会事业。加强公办和普惠性民办幼儿园建设，大力提高办园质量。深入推进义务教育“三大提升工程”，完成中小学品质提升试验区创建。整合市属职业教育资源，组建合肥工业学校。支持合肥学院高水平创建应用型合肥大学。统筹布局四级公共文化服务设施网络，推进乡镇综合文化服务中心和中心村农民文化乐园建设，加快建设数字化图书馆、文化馆、博物馆。广泛开展“大湖名城、悦读合肥”活动。加大文艺精品创作力度，争获全国“五个一”工程奖。推进哲学社会科学创新工程，加强庐剧、巢湖民歌等“非遗”保护和弘扬。正式出版《合肥通史》。积极推动社会力量办医，深入开展名院建设，全面提升医疗服务水平。落实“二孩”生育政策，加大对特殊困难计划生育家庭的帮扶力度。推进城乡公共体育服务一体化，进一步打造环巢湖体育“四季歌”品牌，精心办好环湖国际马拉松、国际铁人三项、第十一届市运会等重大赛事。

   建设平安合肥。启动“七五”普法，全面落实“谁执法、谁普法”责任制。完善公共法律服务体系，扩大法律援助范围。推进立体化、数字化社会治安防控体系建设，强力整治治安突出问题，依法加强网络社会管理，严厉打击各类违法犯罪活动。完善矛盾排查预警和调处化解综合机制，有效预防和化解矛盾纠纷。深化信访工作制度改革，强化重点信访事项督查督办，加强进京非访专项治理，化解一批信访积案。完善人防应急指挥平台体系，深入开展争创社区民防规范化建设“示范点”活动。以开展“铸安”行动为主线，加强基层基础和标准化建设，深化非煤矿山、危险化学品、建筑施工、道路交通、消防等安全生产专项治理。强化食品药品市场监管，创建食品药品安全城市。积极支持军队改革，大力推进国防设施、战备训练、新型民兵力量等建设，普及国防教育，提升征兵质量，全面拓展军民融合深度发展，争创全国双拥模范城“八连冠”。支持工会、共青团、妇联、红十字会等人民团体广泛参与社会管理和公共服务，扎实做好民族宗教、对台侨务、防震减灾、地方志、统计、新闻、档案、保密、社科、仲裁等工作。

   （十）着力推进政府自身建设。

   坚持依法行政。自觉运用法治思维、法治方式谋划和推进政府工作。严格执行人大决议决定，自觉接受市人大及其常委会监督，认真办理人大代表议案及建议、批评和意见。主动接受市政协民主监督，不断提高政协提案办理满意度。广泛听取民主党派、工商联、无党派人士、各人民团体和社会各界的意见，高度重视社会监督和新闻舆论监督。健全完善政府重大决策合法性审查机制和法律顾问制度，严格实行重大决策终身追究制、责任倒查制和行政执法自由裁量权基准制。

   强化执政为民。自觉践行“三严三实”要求，坚持不懈反对“四风”。按照“经济工作项目化、项目工作责任化”的要求，严格执行工作责任制、项目负责制、行政问责制，深入开展“四督四保”行动，确保中央、省委省政府和市委各项决策部署落到实处。大力推进简政放权，进一步减程序、减费用、减时限。创新服务方式，提升各级政务服务中心办事效能，完善“12345”政府服务直通车运行机制。加大公务员培训力度，提升公务员队伍整体素质。健全考核体系，坚决纠正不作为、慢作为、乱作为，严厉惩处失职、渎职。政府全体公务人员特别是各级领导干部要继续发扬“拼”的精神，始终保持“干”的状态，敢于担当，奋发有为，努力以政府的勤奋指数，提升市民的幸福指数、城市的繁荣指数。

   恪守清正廉洁。严守政治纪律和政治规矩，强化党风廉政建设主体责任，落实巡视反馈问题整改措施，始终保持反腐败高压态势，不断提高拒腐防变和抵御风险能力。严格落实中央“八项规定”、国务院“约法三章”和省市相关规定，厉行节约、反对浪费，严控“三公”经费支出。强化行政监察和审计监督，对公共资金、国有资产、国有资源和领导干部履行经济责任情况实行审计全覆盖。深入推进政务公开，确保权力在阳光下运行。

   各位代表！

   “十三五”发展的宏伟蓝图已经绘就。迈上新的历史征程，我们责任重大、使命光荣。让我们紧密团结在以习近平同志为总书记的党中央周围，在省委省政府和市委的坚强领导下，锐意进取，开拓创新，真抓实干，为加快建设长三角世界级城市群副中心、进一步开创“大湖名城、创新高地”建设新局面而不懈奋斗！
